# Murine Models of Acute Pancreatitis: A Critical Appraisal of Clinical Relevance

**DOI:** 10.3390/ijms20112794

**Published:** 2019-06-07

**Authors:** Pedro Silva-Vaz, Ana Margarida Abrantes, Miguel Castelo-Branco, António Gouveia, Maria Filomena Botelho, José Guilherme Tralhão

**Affiliations:** 1 Health Sciences Research Centre, University of Beira Interior (CICS-UBI), 6200-506 Covilhã, Portugal; mcbranco@fcsaude.ubi.pt; 2 General Surgery Department, Unidade Local de Saúde de Castelo Branco, 6000-085 Castelo Branco, Portugal; agouveia@ulscb.min-saude.pt; 3 Faculty of Health Sciences, University of Beira Interior, 6200-506 Covilhã, Portugal; 4 Coimbra Institute for Clinical and Biomedical Research (iCBR) area of Environment Genetics and Oncobiology (CIMAGO), Faculty of Medicine, University of Coimbra, 3000-548 Coimbra, Portugal; mabrantes@fmed.uc.pt (A.M.A.); mfbotelho@fmed.uc.pt (M.F.B.); jglrt@hotmail.com (J.G.T.); 5 Faculty of Medicine, University of Coimbra, 3000-548 Coimbra, Portugal; 6 Biophysics and Biomathematics Institute, IBILI-Faculty of Medicine of University of Coimbra, 3000-548 Coimbra, Portugal; 7 Surgery Department, Centro Hospitalar e Universitário de Coimbra, 3000-075 Coimbra, Portugal

**Keywords:** acute pancreatitis, animal models, murine, experimental models, biomarkers, inflammation

## Abstract

Acute pancreatitis (AP) is a severe disease associated with high morbidity and mortality. Clinical studies can provide some data concerning the etiology, pathophysiology, and outcomes of this disease. However, the study of early events and new targeted therapies cannot be performed on humans due to ethical reasons. Experimental murine models can be used in the understanding of the pancreatic inflammation, because they are able to closely mimic the main features of human AP, namely their histologic glandular changes and distant organ failure. These models continue to be important research tools for the reproduction of the etiological, environmental, and genetic factors associated with the pathogenesis of this inflammatory pathology and the exploration of novel therapeutic options. This review provides an overview of several murine models of AP. Furthermore, special focus is made on the most frequently carried out models, the protocols used, and their advantages and limitations. Finally, examples are provided of the use of these models to improve knowledge of the mechanisms involved in the pathogenesis, identify new biomarkers of severity, and develop new targeted therapies.

## 1. Introduction

Acute pancreatitis (AP), especially severe cases, is a major clinical and financial burden, representing over 320,000 hospital admissions in the United States in 2012 [1]. The incidence of AP ranges from 4.9 to 80 cases per 100,000 persons per year, with equal affinity for each gender [1,2]. The two most common causes of AP are gallstone disease and alcohol [2,3]. The severity and the outcomes are highly variable and unpredictable [4]. Approximately 80% of cases present with mild and interstitial AP. A lower number of patients’ courses with a severe form characterized by the presence of persistent organ failure with local and systemic complications associated with a significant morbidity and mortality [5,6]. Mortality in severe form may reach 30% to 50% [1,6,7]. Unfortunately, the pathophysiology of the AP remains unclear, leading to a lack of effective preventive or therapeutic strategies [8,9].

Evaluating the severity of AP is the major issue that will influence the clinical outcome. Nevertheless, the factors (etiological, environmental, and genetic) that induce severity remain uncertain. The high variability of the severity of AP and the problems in accessing the pancreas in a clinical study have further hindered attempts to clarify its pathophysiology [10]. Therefore, research work using animal models is crucial to increase the understanding of this pathology in order to improve treatments. Since Claude Bernard first debuted the practice in 1856, different animal models have been developed [11], with rodents being the animal most available due to low cost, high reproducibility, mimicking conditions in the human disease [12], and the growing efficiency in manipulating gene structure. An ideal model should be simple and easily reproducible, with the ability to produce controlled severity in order to mimic human disease and answer the experimental question [13]. When performing these models, the researcher should be based on the elevation of pancreatic enzymes, such as amylase and lipase, in order to confirm the induction of AP, taking into account that these enzymes do not reflect the degree of severity. Pancreatitis severity can be assessed by significant histological changes such as interstitial edema, acinar cell death, parenchymal loss, hemorrhage, inflammatory cell infiltration, and vacuolization, and the evaluation of local or systemic complications, with the lung being the organ most involved [14].

In this review, an overview of murine models of AP will be discussed, with special focus on protocols used and their advantages and limitations. Furthermore, examples of the use of these models with the objective of improving the knowledge of the mechanisms involved in the pathogenesis, identifying new biomarkers of severity, and developing new targeted therapies are also provided.

## 2. Applicability of Murine Models to Assess the Severity of Acute Pancreatitis

Banks et al. [6] defined and stratified the severity of AP into three degrees: mild, moderately severe, and severe, based on the absence or presence of persistent organ failure and local or systemic complications. As the severe form is associated with extensive disease characterized by significant pancreatic necrosis and systemic inflammation, which may lead to multiorgan failure and death, the early prediction of severity becomes a major issue in the management of AP.

The severity of AP in murine models is difficult to predict. Nevertheless, some models are associated with mild AP, while others are associated with severe AP (Table 1). The severity of AP is assessed by inflammatory markers and histologic changes. The inflammatory markers most commonly used are protein C-reactive [15], pro-inflammatory cytokines [16,17], tumor necrosis factor-α [18], and procalcitonin [19]. Concerning histological evaluation, Schmidt et al. [20] published the first and most commonly cited scoring system. It uses five parameters: edema, acinar necrosis, hemorrhage and fat necrosis, and inflammation and perivascular infiltrate. Besides pancreatic evaluation (edema, inflammatory infiltration, parenchymal necrosis and hemorrhage), Ding et al. [21] also assessed the liver, kidney, and lung for histological changes. Klopfleisch [22], in his systematic review, analyzed the available histologic scores, concluding that all are based on the Schmidt score having been modified marginally with the omission of a single parameter or including vacuolization as an additional parameter.

However, a severity score related to the Revised Atlanta Classification [6] is not available, to our knowledge, which stratifies murine models of AP in mild, moderately severe, and severe. This tool would be very important to increase the clinical relevance of murine models of AP.

## 3. Murine Models and the Etiology of Acute Pancreatitis

Gallstones remain the most common cause of AP, while up to 25% to 30% of cases can be attributed to alcohol [7]. Despite these two more frequent etiologies, other factors, in 10% of the cases that are described, can influence the severity, such as endoscopic retrograde cholangiopancreatography [23], renal failure [24], diabetes [25], obesity [26], tobacco [27], drugs and toxins [28], genetic factors [29], trauma [30], autoimmune [31], and hyperlipidemia [32].

Since the pathophysiology of this disease is not well understood, it is fundamental to study each cause of AP in order to understand the underlying mechanisms. In this sense, researchers make a great effort to mimic these clinical etiologic factors in murine models. Studies suggest that murine models have successfully characterized intracellular processes that precede tissue injury. 

Due to this, it is important that the researcher has knowledge about each murine model and the clinical etiologic factor that mimics AP to better evaluate the issue that is being addressed (Table 2).

## 4. Murine Models: A Practical Overview

### 4.1. Hormone-Induced or Hyperstimulation Acute Pancreatitis Model 

Normal pancreatic metabolism is associated with physiological concentrations of secretagogues. AP results from an excess availability of secretagogues, which leads to a high secretion of pancreatic digestive enzymes. One of the most frequently used is caerulein, which is a decapeptide cholecystokinin analogue that stimulates pancreatic secretion. This model histologically simulates the early phase of AP in humans [33]. Mild, interstitial, and edematous pancreatitis was developed by Lampel et al. [34] in rats and severe AP with necrosis of acinar cells was developed by Niederau et al. [35] in mice. The continuous infusion of supramaximal caerulein can be administrated intravenously [34,36,37], subcutaneously [38,39], or intraperitoneally [40], with intravenous being the best way of administering the hormone to rats and mice. However, it is not commonly performed due to the requirement of vascular cannulation and anesthesia. This method was modified via intraperitoneal in the lower left or right quadrant of the abdomen. Between one and 12 doses may be given hourly, via subcutaneous or intraperitoneal, to induce AP. Table 3 summarizes the different routes of administration of caerulein, as well as the most commonly used doses. Histologically interstitial edema develops one hour after the infusion, with a maximum after 12 h [34]. This model has been useful for the evaluation of the AP by analyzing the pathophysiology [41,42,43,44], severity [45,46,47,48,49], target therapies [50,51,52,53,54], course, outcomes [55], and related pulmonary [56,57] and cardiac [58] injuries. It also mimics the pathophysiology of AP caused by scorpion venom [59] and cholinergic toxins [60] in humans. The exact mechanism by which caerulein induces disease is not totally understood. Studies have shown that caerulein causes an abnormal localization of the zymogen and lysosomal hydrolases that are activated intracellularly within the acinar cells [61]. In its turn, lysosomal cysteine protease cathepsin B appears to be an important factor in the activation of trypsinogen to trypsin [62]. This model has also been used in the study of the identification of new protein alterations and biomarkers [63] characterizing pancreatic inflammatory damage with proteomic [64] and metabolomic [65] analysis. The advantages of this model are its noninvasiveness, inexpensiveness, rapid induction, and wide reproducibility and applicability. It could also be applied to in vitro research [66], and also be used for evaluating systemic disease progression, since it is an important tool for researching the pulmonary involvement of AP [67,68]. The major disadvantage is that only a mild form is developed, and the clinical relevance is limited.

### 4.2. Alcohol-Induced Acute Pancreatitis Model

One of the major etiologic factors of AP is alcohol. However, it is well documented that AP induced by alcohol alone has been difficult to achieve [81,82]. Foitzik et al. [83] developed a study to evaluate the factors thought to be involved in the pathogenesis of AP associated with alcohol, since alcohol causes pancreatic injury only when combined with other factors such as exocrine hyperstimulation. The most commonly used are secretagogues [82], lipopolysaccharides [84], or palmitoleic acid [70,85]. This model has been used to analyze the effects of alcohol on pancreatic microcirculation, regeneration, and the role of oxidative stress [86]. The alcohol-induced model could be performed via intravenous [71], oral [69,71], intraperitoneal [70], and intraductal [72] administration. Table 3 summarizes the different routes of administration of alcohol, as well as the most commonly used doses. Huang et al. [70] studied the relative importance of oxidative and non-oxidative pathways in mitochondrial dysfunction, pancreatic damage, and the development of alcoholic AP, and whether the deleterious effects of non-oxidative metabolism of alcohol are preventable. This study enabled researching potential specific treatments via the inhibition of the generation of fatty acid ethyl esters. Kiziler at al. [72] measured markers of oxidative damage in pancreatic tissue, studying how alcohol injures pancreatic parenchyma tissue. They induced AP by the injection of ethyl alcohol into the common biliopancreatic duct. The authors concluded that iron in serum and pancreatic tissue in rats with early-stage AP was associated with several microvascular changes and oxidative pancreatic injury. Schneider et al. [71] showed that the intravenous alcohol-induced pancreatitis offers a valid model. Studies have suggested that alcohol is related with an increase in ischemia damage and have shown the direct damage of the pancreas [87]. This model is relatively simple and can be performed at a low cost. The major disadvantage is the difficulty in reproducing it and its low clinical relevance. The failure to reproduce acute pancreatitis reflects the human condition, and it is necessary to take into account an additional sensitizing factor or a genetic predisposition regarding the development of alcoholic AP.

### 4.3. Gene Knockout Acute Pancreatitis Model

The gene knockout models played a key role in understanding the relevance of genetic factors in AP. A knockout mouse model provides a more efficient way to study the impact of complete loss of function or the deletion of a gene during disease initiation and progression. The process of targeting gene provides the ability to alter a specific gene in order to better discern its biological role [88]. Mice are currently the most closely related animal to humans, because both species share about 99% of the same genes [89], for which this technique can easily be applied. This animal has the ability to obtain real totipotent embryonic stem cells [90]. Gene knockout in rats is much harder, and has only been possible since 2003 [91]. These models are usually used to evaluate the pathogenesis and outcomes of AP [69]. Venglovecz et al. [43] using aquaporin 1 knockout mice, concluded that aquaporin 1 plays an essential role in pancreatic ductal fluid and bicarbonate secretion, which probably contributes to the increased susceptibility of pancreatic inflammation. Tao et al. [92] studied the role of β-arrestins, which are the regulators and mediators of a G protein-coupled receptor signaling, in pancreatic inflammation. They observed that β-arrestin 1 alleviates AP, and it may be used as a potential therapeutic target. Norkina et al. [93] studied the involvement of the Reg/PAP cell stress gene in the protection or recovery from pancreatic injury. They concluded that Reg/PAP cell stress genes may be protective due to their anti-apoptotic activity. Jancsó et al. [94] used CTRB1-deficient mice to study the role of chymotrypsin in the early phase of AP. They concluded that CTRB1 protects against secretagogue-induced pancreatitis by reducing trypsin activity. This study highlights the role of protease inhibitors in target therapies of AP. The gene knockout models are also an important tool to study the pancreatic inflammation changes and characterized multiorgan failure [95,96]. The gene knockout model is expensive and complex; its major advantage is that by deleting the specific gene under study, its specific function or effect could be analyzed more efficiently.

### 4.4. Nutrient-Induced Acute Pancreatitis Model

AP induced in rats treated with ethionine was described by Faber and Popper [97]. This model has frequently been used to study the pathogenesis of AP and histologic changes of pancreatic parenchymal cells. Lombardi et al. [98] observed hemorrhagic AP with massive fat necrosis in the peritoneal cavity with a total mortality of mice after a choline-deficient ethionine diet. Although the molecular mechanism remains unclear, a choline-deficient ethionine diet and arginine or other basic amino acids cause severe AP associated with a high mortality [99]. They also showed that a choline-deficient diet without ethionine did not cause pancreatic inflammation or mortality [98]. Guilliland and Steer [100] changed the original model, allowing the gradation of AP severity and mortality in order to further study the outcomes of this disease. The choline-deficient ethionine diet model is widely used since it is a noninvasive model, avoiding exogeneous shock. This model was used to evaluate the involvement of pancreatic stellate cells in the development of fibrosis associated to AP [101], and has been used recently to evaluate the signaling pathway that promotes inflammation [96] and new target therapies [102]. The induction of AP can also be achieved with arginine. Mizunuma et al. [103] proposed a model that established the intraperitoneal route with L-arginine hydrochloride. Several modifications of this model showed that higher doses can produce high mortality, repeated doses promote necrosis, and reduced doses may delay the time of onset of AP [73,74,104]. Table 3 summarizes the most commonly used doses of L-arginine when administered by the intraperitoneal route. A histological examination revealed degenerative changes to intracellular organelles and the nuclei of acinar cells [105]. Uçmak et al. [106] induced AP with an L-arginine model to investigate the potential effect of silybin, which is a potent antioxidant. They verified that silybin, when used in a prophylactic rather, ameliorates serum oxidative stress parameters. The advantage of this model is its high reproducibility and applicability for researching the different phases of AP and evaluating distant organ injury. The major disadvantage of the arginine-induced AP model is its low clinical relevance, since only two clinical cases of arginine AP in humans are described [107,108].

### 4.5. Closed Duodenal Loop Acute Pancreatitis Model

**Figure 1:**
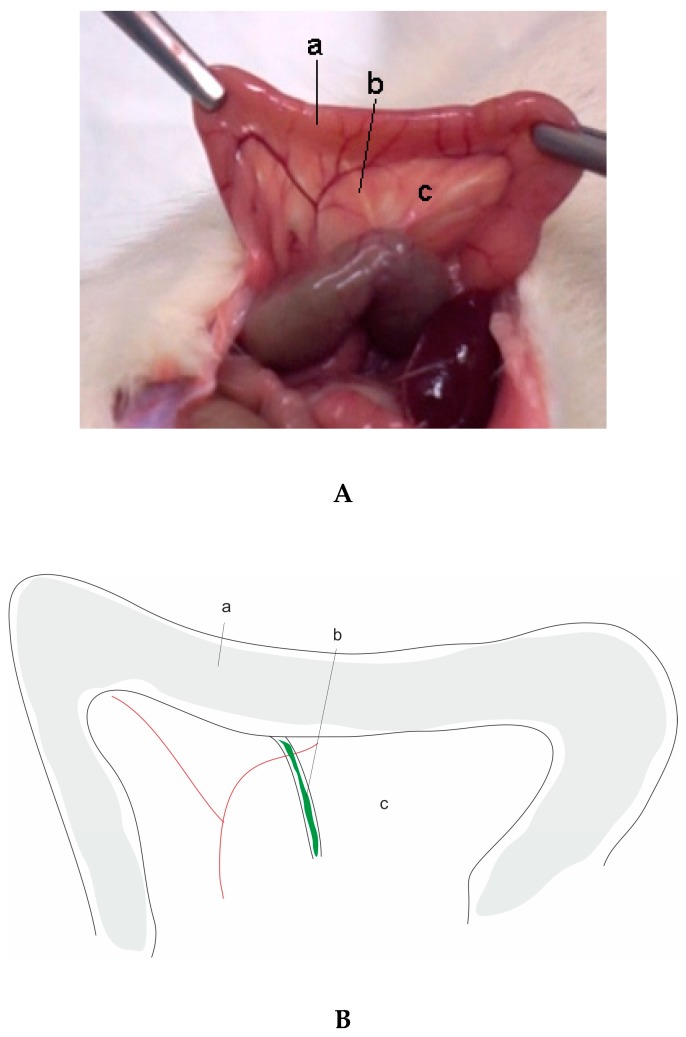
(**A**) Rat pancreas anatomy. Image: The pancreas of an adult rat showing the duodenum and common biliopancreatic duct. Rodent pancreas is soft and diffuse compared with the human pancreas. Photo provided by authors lab. a- duodenum; b- common biliopancreatic duct; c- pancreas. (**B**) Schematic showing the anatomy of rat pancreas. Image: picture showing the schematic anatomy of the pancreas, duodenum, and common biliopancreatic duct. a- duodenum; b- common biliopancreatic duct; c- pancreas.

**Figure 2:**
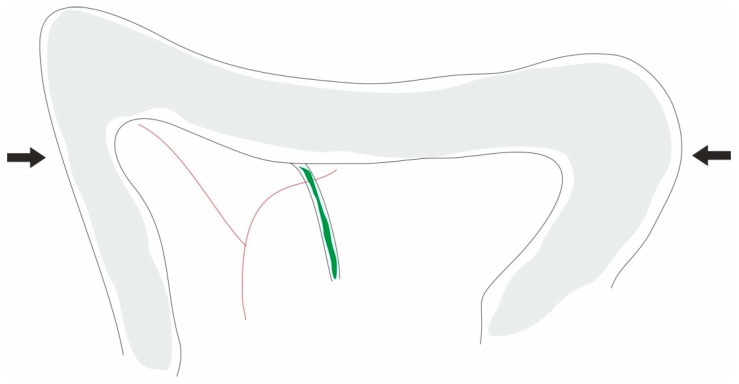
Closed duodenal loop acute pancreatitis model. Image: picture showing the location of the closed loop of the duodenum. According to aseptic techniques, the duodenum is exposed by a laparotomy, the common biliopancreatic duct is identified, and the duodenum is obstructed by the placement of two ligatures: one just beyond the pylorus—that is, proximally to the duct—and the second placed distally to the duct (arrows).

In a normal physiological environment, verifying a pressure gradient between the common bile duct and duodenum prevents the duodenopancreatic reflux. The closed duodenal loop model, by changing this normal condition, increases intraduodenal luminal pressure, causing reflux and leading to AP. This model is characterized by the ligation of the duodenum proximal and distal to the union of the common biliopancreatic duct (Figure 1A,B and Figure 2) [109]. The histopathological changes verified with this model consist of intralobular edema, and progressively hemorrhagic pancreatic necrosis [110]. This model is more suitable for the study of pancreatic necrosis as well as for the study of new therapies [110]. Adler, Kern, and Scheele [111] proposed the cannulation of the bile duct to divert the bile away from the duodenum and avoid the involvement of bile in the ensuing pancreatitis, and bile is normally diverted into the jejunum. Several researchers modified this model [112], producing necrohemorrhagic AP. These findings support that the reflux of the duodenal contents into the pancreatic duct is an important mechanism in the development of AP. Other authors [113] instill infected bile or bile sterilized into the duodenum under pressure, producing severe AP. Since this model is associated with a high mortality of the rats, some researchers modified the original model to a temporary ligation of the duodenum [114]. This model is also suitable for the study of bacterial translocation during AP, since the discontinuation of the duodenum leads to functional changes in the mucosal barrier, causing small bowel bacterial overgrowth [115,116]. Sugimoto et al. [117] developed a model of reversible pancreatitis, with an incomplete closed duodenal loop model. They demonstrated that the damage of microcirculation due to tissue ischemia played a role in the increasing severity of AP. This model clarified the role of microcirculation impairment in the pathogenesis of AP, allowing for the research of targeted therapies. The advantage is its simplicity, reproducibility, and use on small animals such as rats. However, the need for surgical intervention, the increased pressure levels verified in the closed duodenal compartment, and the pancreatic duct, as well as the controversial role of bacterial infection, make this model not widely used in scientific research. However, this model has clinical relevance, since there are cases of AP associated with duodenal obstruction in medical literature [118].

### 4.6. Biliopancreatic Duct Injection Acute Pancreatitis Model

**Figure 3:**
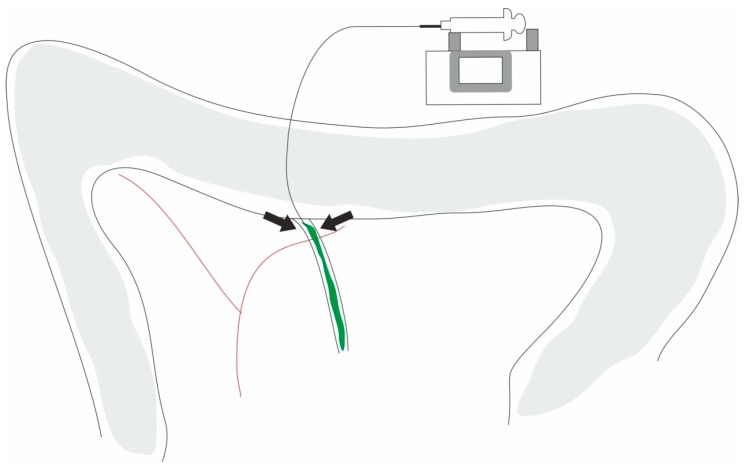
Biliopancreatic duct injection acute pancreatitis model. Image: picture showing the retrograde ductal infusion technique. According to aseptic techniques, the duodenum is exposed by a laparotomy, and the common biliopancreatic duct is identified and cannulated; after the retrograde infusion, the duct is ligated (arrows).

The dissection of the pancreatic duct and its cannulation provides an alternative way of inducing an experimental AP model. The original model was developed in cats, in 1979, by Reber et al. [119]. Several modifications were made in order to reduce the degree of technical difficulty. The most common surgical technique (Figure 3) consists of a duodenotomy, which is a retrograde injection of bile salts (with or without activated pancreatic enzymes) into the pancreatic duct at the ampulla that leads to severe AP [120]. To induce AP, some compounds infused into the pancreatic duct are used, such as sodium glycodeoxycholic [20] and sodium taurocholate [78,79,121,122,123] being the last the most used, because it is thought to be the one that most resembles clinical biliary pancreatitis [124]. This bile salt can also be used to induce biliary AP with multiple organ failure. The severity of AP can be obtained by changing either the pressure or the concentration of the bile salt that is used. The severe form is characterized by edema, necrosis, and hemorrhage. The infusion of solution sodium taurocholate induced acute hemorrhagic pancreatitis with mortality range between 24–100% [75,76,77,80,125,126]. Table 3 summarizes the different doses of sodium taurocholate that are most commonly used. This model is appropriate to study both local and systemic complications of AP, and it can be used to study target therapies. To improve its limitations, this model was changed by combining low-dose intraductal glycodeoxycholic acid with intravenous caerulein [20]. The severity achieved with this model is similar to human disease, and for this reason, it could be used for the study of AP pathogenesis [127,128] and target therapies [129]. This is an easy and reproducible model for severe AP. However, an important limitation is its clinical and pathogenic relevance due to the questionable role of duodenal reflux in the pathogenesis of AP. Another disadvantage is the impossibility of quantifying the pressure with which the solution is applied to cause AP.

### 4.7. Vascular-Induced Acute Pancreatitis Model

Pancreatic microcirculatory failure is the major cause of mortality in severe AP, and is thought to be crucial in the early events of AP with multiple organ dysfunction syndrome [130]. The decrease of pancreatic microcirculatory blood flow volume and velocity, and the increase of microvascular permeability lead to pancreas edema and inflammatory infiltration in the early phase of AP [131]. In 1962, Pfeffer et al. [132] found that different degrees of occlusion of the pancreatic microcirculation cause different pancreatic changes, from edema to acute hemorrhagic, necrotizing pancreatitis. This is particularly useful in the study of coagulopathy and the thrombosis of microvessels [133]. Liu et al. [134] studied the effect of vascular bradykinin on pancreatic microcirculation in rats with severe AP. They concluded that vascular bradykinin can improve pancreatic microcirculation. In addition, several studies have been performed to occlude pancreatic arteries [135] and veins [136]. The advantage of the vascular model is that it is relatively inexpensive, although it does not reliably induce the same degree of severity of AP. However, it is important for studying microcirculatory disorders such as coagulopathy or microvascular thrombosis. The disadvantages are the dramatic surgical trauma exerted upon the animals, the need for a tight protocol, and specific surgical resources.

### 4.8. Ischemia/Reperfusion Acute Pancreatitis Model

The role of ischemic injury as a cause of AP is well known. Some studies have shown a correlation between the impairment of pancreatic microcirculation and the degree of ischemic injury. Pancreatic microcirculation is the target of reperfusion injury after ischemic. Hoffman et al. [137] developed a model of complete ischemic/reperfusion of the pancreas in rat, causing pancreatic microvascular failure. The severity of changes depends on the duration of ischemia and reperfusion. Dembinski et al. [138] induced AP by clamping the inferior splenic artery for 30 minutes. They induced necrotizing AP with subsequent regeneration within a few weeks. The ischemic/reperfusion model has been used for the study of target therapies such as obestatin [139] and ghrelin [140] and for the study of several biomarkers [141]. The major disadvantages of this model include its reproducibility, being incomplete ischemia, and the inability to measure the remaining pancreatic blood supply. The irreversible ischemia makes this model unsuitable for reperfusion studies. Furthermore, it is difficult to achieve the quantitative analysis of the post-ischemic reperfusion failure.

### 4.9. Duct Ligation Acute Pancreatitis Model

**Figure 4:**
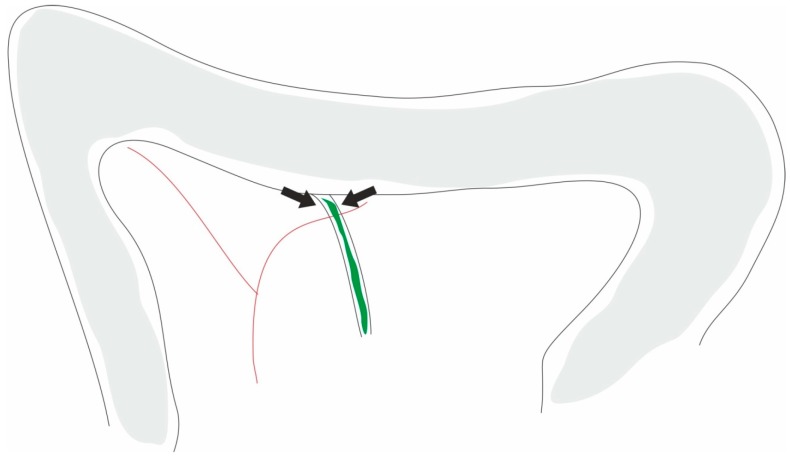
Image: picture showing the site of ligation of the common biliopancreatic duct in the rat. According to aseptic techniques, the duodenum is exposed by a laparotomy; the common biliopancreatic duct is identified and ligated at the level of the duodenum (arrows).

AP may be induced by ligating the distal bile duct at the level of the duodenum (Figure 4). The first report that associated this model to changes in the exocrine function of the pancreas refers to Churg et al. [142]. This model was used by several researchers to study the pathogenesis [143] and target therapies [144,145], and was developed in an attempt to mimic the clinical situation of a gallstone that obstructed the ampullary orifice with the consequent reflux of bile into the pancreatic duct that lead to AP. In rats, the initial changes such as edema, inflammation, and hyperamylasemia are compatible with AP [146]. This model also induces obstructive hepatic cholestasis and hepatic cholangitis in rats. However, in order to improve this model, some changes were made, namely the combination of pancreatic duct ligation and secretory stimulation, such as caerulein or sodium taurocholate. Studies have shown that in the duct ligation model in rats, the predominant mechanism of cell death is apoptosis. This model may be suitable for the study of bacterial translocation, since the obstruction of bile flow into the intestine causes small bowel bacterial overgrowth [116]. The exclusion of the pancreatic proteases in the gut lumen also alters the intestinal permeability [147]. The advantage of the duct ligation model is that it avoids artificial drug usage, which may produce unwanted systemic effects, as well as the theory relating to clinical biliary AP with biliary pancreatic reflux. However, this is a complex and technically difficult model, and has an associated high cost.

## 5. Clinical Relevance of the Models and Future Directions

Personalized medicine has become a major research topic in the scientific community. This approach will, in the patient’s prolonged life, improve their quality in the future, bringing new challenges to health care. This prolonged lifetime will increase the incidence of AP as well as more severe forms associated with the patient’s clinical condition and its comorbidities. This scenario makes the deepening knowledge of the pathophysiology of AP extremely important. Although the clinical relevance of murine models of AP is a controversial subject, they have contributed greatly to the elucidation of the pathophysiology of this important disease.

The clinical presentation of AP is very variable, from mild to moderately severe and severe, with the latter characterized by the persistence of multiorgan failure [6]. Severe AP associated with infected necrosis represents a very high morbidity and mortality. The actual management of severe AP is based on intravenous fluid therapy [148,149], pain control [150], and adequate nutrition [151], utilizing all of these is the best way to prevent early deaths. In cases of infected necrotizing pancreatitis, an endoscopic or surgical step-up approach is evaluated according to each patient and clinical condition [152]. The complexity of AP in human patients is very high, making it difficult for any of the murine animal models available to develop the disease with all the features comparable to human disease. This is true: since predicting the AP severity of each model is difficult, most studies do not stratify animals into moderately severe forms, and there is no severity histologic score that stratifies animals into the three degrees of AP severity. This issue is of extreme importance, since it will allow a more adequate translation of outcomes, and will influence the methodology adopted in each study in the context of the question being addressed.

However, murine models will continue to be an indispensable tool for the study of AP; they will also improve the outcomes of this condition in humans, not only in the field of prevention, but also in attempting new targeted therapies. Unfortunately, most of the results achieved in these models are not confirmed when translated to human studies.

The choice of the best model is fundamental, and should be based either on the etiology or on the risk factors that might modulate disease severity, always keeping in mind the goal of the research.

## 6. Conclusions

In order to circumvent the limitations and maximize the advantages of each murine model, improvements and combinations of the models have been made to reproduce the disease in humans. These models should attempt to replicate the mechanisms and processes underlying the disease, examine therapeutic interventions, and analyze basic characteristics of acute injury, inflammation, or tissue reconstitution. However, at the present time, none of the existing murine models of AP are totally acceptable. Murine models have considerably contributed to the understanding of the pathophysiologic mechanisms of AP. Therefore, further elaboration of protocols is needed to improve and facilitate the choice of a specific model for a specific question. In this sense, it is essential to invest in the translational research, since these models are an important tool for improving medical care and outcomes for patients with AP.

## Figures and Tables

**Table 1:** Animal models for acute pancreatitis according to the severity degree.

AP Classification (According Revised Atlanta Classification [6])	Animals	Models
Mild acute pancreatitis - No organ failure - No local or systemic complications	Rats	Hormone-induced model
Moderately severe acute pancreatitis - Organ failure that resolves within 48 h - Local or systemic complications without persistent organ failure		-
Severe acute pancreatitis - Persistent organ failure (>48 h) - Single organ failure - Multiple organ failure	Mice	Hormone-induced model
	Mice and Rats	Closed duodenal loop model *
	Mice	Alcohol-induced model *
	Mice and Rats	Nutrient-induced model
	Mice and Rats	Biliopancreatic duct injection model
	Mice and Rats	Vascular-induced model
	Mice and Rats	Ischemia/Reperfusion model *
	Mice and Rats	Duct ligation model *

The severity of AP is a very important issue for the correct approach of this disease. The choice of the model and animal is crucial for the correct design and answer to the question under study. * In several studies, the AP severity is very variable.

**Table 2:** Animal models for acute pancreatitis according to the etiology and factors.

	Animals	Models
Etiology	Mice and rats	*Biliary pancreatitis* Biliopancreatic duct injection model Duct ligation model *Alcoholic pancreatitis* Alcohol-induced model
Factors	Mice and rats	*Bacterial translocation* Closed duodenal loop model Duct ligation model *Microcirculation impairment* Vascular-induced model Ischemia/Reperfusion model *Cholinergic agents* Hormone-induced model *Diets* Nutrient-induced model *Genetic* Gene knockout model *Trauma* Ischemia/Reperfusion model Closed duodenal loop model

Murine models are most commonly used to study AP. In mice and rats, AP (acute inflammation with necrosis and hemorrhage when severe) can be induced by injections of caerulein, alcohol, bile salt infusion, duct ligation, several nutrients such as choline-deficient ethionine-supplemented diet and L-arginine, closed duodenal loop, alterations in genetic animal structure, and changes of pancreatic vascular irrigation. Whether these models produce all the characteristics of human AP remains unclear.

**Table 3:** Protocols of the most used acute pancreatitis (AP) models in mice and rats. BW: body weight.

AP Model	Animals	Protocols		References	Clinical Relevance
		Administration Route	Doses		
**caerulein**	**mice**	**Intravenous**	6 h continuous infusion of 100 µg/kg/h	[37]	- Relevant to understanding the early AP mechanisms - Pulmonary injury mimics the respiratory involvement in humans - Structural changes of acinar cells are similar to human AP - Preserves acinar physiology throughout the experimental disease course - Mimics the pathophysiology of AP caused by scorpion venom and cholinergic toxins in humans
		subcutaneous	7 h of injections at 50 µg/kg	[39]	
		intraperitoneal	8 h of injections of 10 µg/mL, 0.2 mL/mouse) over two consecutive days	[40]	
			7 h of injections at 50 µg/kg	[44,45]	
			50 µg/kg every two hours for five rounds	[42]	
			10 h of injections at 50 µg/kg	[41,43]	
	rats	intravenous	5 µg/kg/h for periods up to 24 h	[34]	
			3–h continuous infusion of 7.5 µg/kg/h (7.5 µg/kg/h × 3 h)	[36]	
		subcutaneous	5 µg/kg/h for 3 h (hourly injection)	[38]	
			Four injections of 20 µg/kg/h hourly	[47,51]	
			Injection of 10 µg/kg	[50]	
		intraperitoneal	Two injections of 40 µg/kg at hourly intervals	[46]	
alcohol	mice	oral or intragastric	Single intragastric dose of ethanol (6.0 g/kg BW) in NRF2-KO mice	[69]	- Poor clinical relevance
		intraperitoneal	Two intraperitoneal injections of ethanol (1.32 g/kg BW) and palmitoleic acid (1.5 mg/kg BW), separated by one hour	[70]	
	rats	intravenous	Bolus of 2 g/kg BW followed by continuous IV alcohol application of 0.365 g/kg BW/h with an additional 3 mL/kg BW saline solution	[71]	
		oral or intragastric	Intragastric bolus of ethanol 2.3 g/kg BW followed by the continuous infusion of 0.365 g/kg BW/h IV	[71]	
		intraductal	Injection of 48% ethyl alcohol in a volume of 1 cm^3^ into the common biliary duct	[72]	
L-arginine	rats	intraperitoneal	2-h injections of 8%	[73]	- Mimics the circulatory, respiratory, and renal alterations that occur in human AP
			250–500 mg/100 g BW	[74]	
Duct infusion-induced model	mice	sodium taurocholate	10 µL/min for 5 min of 2.5–5%	[75]	- Clinical relevance is unclear
	rats	sodium taurocholate	5–10 mM with caerulein intravenous 5 µg/kg/h for 6 h	[76,77]	
			1 mL/kg of 3% injected over a 60-second period	[78,79,80]	

The most used protocols in AP animal models are described in the sense of helping those who intend to work in murine models. The potential of combining the existing models in the genetically modified murine animals will improve the knowledge of the pathophysiology process underlying AP.
